# Young Children Intuitively Divide Before They Recognize the Division Symbol

**DOI:** 10.3389/fnhum.2022.752190

**Published:** 2022-02-25

**Authors:** Emily Szkudlarek, Haobai Zhang, Nicholas K. DeWind, Elizabeth M. Brannon

**Affiliations:** Department of Psychology, University of Pennsylvania, Philadelphia, PA, United States

**Keywords:** mathematical ability, number sense, division, arithmetic, approximate number system, approximate arithmetic

## Abstract

Children bring intuitive arithmetic knowledge to the classroom before formal instruction in mathematics begins. For example, children can use their number sense to add, subtract, compare ratios, and even perform scaling operations that increase or decrease a set of dots by a factor of 2 or 4. However, it is currently unknown whether children can engage in a true division operation before formal mathematical instruction. Here we examined the ability of 6- to 9-year-old children and college students to perform symbolic and non-symbolic approximate division. Subjects were presented with non-symbolic (dot array) or symbolic (Arabic numeral) dividends ranging from 32 to 185, and non-symbolic divisors ranging from 2 to 8. Subjects compared their imagined quotient to a visible target quantity. Both children (Experiment 1 *N* = 89, Experiment 2 *N* = 42) and adults (Experiment 3 *N* = 87) were successful at the approximate division tasks in both dots and numeral formats. This was true even among the subset of children that could not recognize the division symbol or solve simple division equations, suggesting intuitive division ability precedes formal division instruction. For both children and adults, the ability to divide non-symbolically mediated the relation between Approximate Number System (ANS) acuity and symbolic math performance, suggesting that the ability to calculate non-symbolically may be a mechanism of the relation between ANS acuity and symbolic math. Our findings highlight the intuitive arithmetic abilities children possess before formal math instruction.

## Introduction

Arithmetic skills underlie the entire elementary school math curriculum (National Governors Association Center for Best Practices, and Council of Chief State School Officers, 2019). Mastery of early arithmetic begins a cascade that unlocks the opportunity to study more advanced branches of mathematics such as algebra, geometry and calculus. According to the US Common Core Standards children learn arithmetic operations in a sequence starting with addition and subtraction, then multiplication, and finally division beginning in grade 3. Division is commonly introduced as the inverse of multiplication, and children’s early understanding of division is mediated via multiplication. Only later in more advanced math education do these representations diverge (Campbell, 1997; Mauro et al., 2003). There is neural and behavioral evidence that division remains more effortful than the other basic arithmetic operations even into adulthood (Ischebeck et al., 2009; Rosenberg-Lee et al., 2011). These findings suggest that division is the most difficult of the four basic arithmetic operations. However, this greater difficultly may be a function of how formal division is taught and not a fundamental aspect of the operation.

Children have some basic intuitions about division before they formally learn how to divide. These basic intuitions may derive from insights into practical mathematics in the world around them, called intuitive action schemas (Riley, 1984; Jitendra and Hoff, 1996; Correa et al., 1998). One hypothesized action schema that supports division is children’s knowledge of how to fairly distribute items amongst people (Blake and McAuliffe, 2011; Shaw and Olson, 2012; Sheskin et al., 2016; Hamamouche et al., 2020).

Another way in which children may begin to form a concept of division is through many-to-one counting to solve multiplication and division word problems. For example, when kindergarteners were presented with the problem “Tad has 15 guppies. He put 3 guppies in each jar. How many jars did Tad put guppies in?” children demonstrated many-to-one counting by counting out 15 guppies into groups of 3, and then counting the number of groups (Carpenter et al., 1993). However, these strategies usually require external support and small set sizes.

A third way children could develop an intuitive sense of division is through experience with their non-symbolic sense of number. The Approximate Number System (ANS) allows children to approximately represent, compare, estimate, and calculate with large sets of objects (Feigenson et al., 2004). A substantial body of work demonstrates that adults, children, infants, and non-human primates can use ANS representations to add and subtract arrays of objects (McCrink and Wynn, 2004; Pica et al., 2004; Barth et al., 2005, 2006; Knops et al., 2009; McNeil et al., 2011; Gunderson et al., 2012; Pinheiro-Chagas et al., 2014; Xenidou-Dervou et al., 2014; Cantlon et al., 2015). Young children can also perform scaling operations on large arrays of objects and multi-step operations (Barth et al., 2009; McCrink and Spelke, 2010, 2016; McCrink et al., 2013, 2016). By using their ANS, children can even solve addend unknown algebra problems (Kibbe and Feigenson, 2015, 2017), and compare ratios of discrete sets of items (Falk et al., 2012). This work indicates that ANS representations can be used in a variety of non-symbolic and approximate mathematical contexts.

It is still an open question whether children can use their ANS to compute a true non-symbolic, approximate division operation. In the approximate scaling task used by McCrink and colleagues a child saw a large set of items which were then hidden behind a white box. A ‘dividing wand’ appeared on top of the box and the child was told “Look! They’re getting divided”. During training, the child watched the dividing wand halve (or quarter in another experiment) a set of objects. During testing, the child compared their imagined quotient to a target set of objects and picked the larger set. Children’s accuracy varied as a function of the ratio between the halved or quartered array and the visible target array, a hallmark of ANS representations. There are two ways in which this scaling task differs from a non-symbolic, approximate division task. First, scaling operations are a specific case of a division operation where the divisor is held constant. A true division operation requires both the dividend and divisor to hold multiple values. Second, this task is not entirely non-symbolic because there is a specific one-to-one correspondence between the ‘dividing wand’ symbol and a given divisor. Thus, it is unknown whether children can use their non-symbolic, approximate sense of number to perform non-symbolic division.

The first goal of the current experiment was to determine whether young children can intuitively divide large quantities with their ANS. To answer this question, we developed a novel non-symbolic division paradigm where both the dividend and the divisor are non-symbolic quantities that vary from trial to trial. Using multiple divisors within one subject allowed us to ask whether children truly have an intuitive sense of division, or whether children are limited to the halving or quartering operations demonstrated previously (McCrink and Spelke, 2010, 2016). To determine whether intuitive division operates over ANS representations, we tested whether accuracy on our non-symbolic division task was dependent on the ratio between the quotient and a target comparison value. Ratio dependent accuracy is a hallmark of the ANS (Feigenson et al., 2004). As a stronger test of our hypothesis, we also independently measured each child’s ANS acuity using a dot comparison task and examined the correlation between intuitive division accuracy and ANS acuity. If children indeed use their ANS to perform approximate division, children with better ANS acuity should perform more accurately on our approximate division task.

As a further test of children’s intuitive division competence, participants also completed a *symbolic*, approximate division task. This task was animated in the same way as the non-symbolic, approximate division task; however, the dot-array dividends and targets were replaced with numerals. Previous research demonstrates that children can perform symbolic, approximate addition and subtraction (Gilmore et al., 2007), mixed symbolic to non-symbolic ratio comparisons (Kalra et al., 2020) and fully symbolic, approximate ratio comparisons (Szkudlarek and Brannon, 2021) before formal instruction. Successful performance on our symbolic, approximate division task would indicate that intuitive division performance is not specific to the numerical magnitude representation afforded by dot arrays, but rather to numerical magnitude representation. Thus, in the current experiment we test whether children’s intuitive division abilities can extend to symbolic division.

Our second goal was to explore how approximate division skill relates to formal teaching about the division operation. If non-symbolic and symbolic approximate division tasks have any use in pedagogical context, they may be most helpful before formal division teaching begins. Our sample included children aged 6 to 9, which spans the age range before and during the beginning of formal division instruction. To ensure that children’s intuitive large number division skill was not dependent on prior instruction about the division operation, we quantified children’s level of symbolic division knowledge with a test of their symbolic, exact division skill. Then, we tested whether children can successfully approximately divide before they have formal knowledge of division as a math operation.

The third and final goal of the current experiment was to examine whether intuitive division skill provides a link between ANS acuity and formal mathematics. Prior work has demonstrated that ANS acuity and symbolic math performance are correlated in children and adults (Chen and Li, 2014; Fazio et al., 2014; Schneider et al., 2016). However, recent findings suggest that performing a mathematical operation non-symbolically and approximately may be a better predictor of symbolic math ability than ANS acuity in both children and adults (Pinheiro-Chagas et al., 2014; Matthews et al., 2016; Starr et al., 2016; Szkudlarek and Brannon, 2021). In the context of the current experiment, sharper ANS acuity may allow for better non-symbolic division calculation. In turn, better non-symbolic division ability may provide students stronger conceptual models of division operations. This stronger concept of division may lead to a sturdier ability to tackle symbolic, exact division calculation in the classroom. Accordingly, we predict a significant correlation between non-symbolic division accuracy and formal mathematical skill, as measured with the Key-Math-3 Numeration test (Connolly, 2007). Furthermore, we predict that intuitive division accuracy will mediate the correlation between ANS acuity and performance on the Key-Math-3 Numeration test. We tested this mediation hypothesis in both children and university undergraduates. Our mediation hypothesis is particularly interesting for adults because there is currently a lack of theorized mechanisms for why the relation between ANS acuity and symbolic mathematics persists into adulthood. For example, the theory that sharper ANS acuity promotes the initial learning of number words does not explain why ANS acuity would still be linked to math skills in adulthood (Odic et al., 2015). If the ability to model arithmetic operations using the ANS is a mechanism of the link to symbolic math, sharper ANS acuity could indirectly impact complex math abilities later in development, opening up further paths of inquiry to explore this relation.

We explored intuitive division ability across three Experiments. In Experiment 1, 6-9 year old children completed non-symbolic and symbolic division tasks and measures of ANS acuity and formal math to examine whether children could successfully perform intuitive division before formal knowledge of the division operation, and whether this ability could serve as a pathway between ANS acuity and symbolic math ability. In Experiment 2, we tested a new cohort of 6-9 year old children on the division tasks using different numerical values to rule out alternative calculation strategies and to replicate the finding that children can perform intuitive division before formal division knowledge. In Experiment 3, we examined university undergraduate’s ability to perform intuitive division, and whether this ability can continue to provide a bridge between ANS acuity and formal math ability into adulthood.

## Materials and Methods

### Child Experiments

#### Subjects

Eighty-nine 6-9 year-old children participated in Experiment 1 (mean age = 7.9 years-old, standard deviation = 1.1 years; 50 female, 39 male). Written parental consent was collected in accordance with a protocol accepted by the University of [blinded] Institutional Review Board. Thirty-two additional children were consented but did not complete both the non-symbolic and symbolic division tasks due to absence and were excluded. The parents of 88 children in the sample completed a detailed demographics questionnaire. 86% identified as Black or African American, 10% as White, 2% as Asian, and 2% as more than one race. A large proportion of children came from families with household incomes of $50,000 or less (7% $150,000+, 6% $150,000 - $100,000, 4% $75,000 - $50,000, 45% $50,000 - $25,000, 30% $25,0000 - $0, and 8% chose not to report). All subjects were recruited from six after school programs in the Philadelphia, PA area. A subset of the children who completed both the non-symbolic and symbolic division tasks completed additional assessments (Dot comparison, *n* = 84; Key-Math Numeration subtest, *n* = 89; Division knowledge assessment, *n* = 82; the Woodcock-Johnson Basic Reading Skills cluster, *n* = 77; and a measure of numeral identification, *n* = 80). All participants received a small toy as a thank you gift.

To replicate our results and ensure that children were indeed performing approximate division to solve our division tasks, forty-two children (mean age = 7.9, standard deviation = 1.2 years, 16 female, 12 male, 14 did not report) were tested in Experiment 2 on the same division tasks with different numerical values from Experiment 1. None of the children participated in Experiment 1. The parents of twenty-five participants completed our demographic form. 56% identified as Black or African American, 12% identified as White, 4% as more than one race, and 28% chose not to report. The majority of children came from households with incomes of $50,000 or less (8% $100,000+, 12% $75,000 - $50,000, 32% $50,000 - $25,000, 8% $25,0000 - $0, and 40% chose not to report). Children in Experiment 2 were tested on the non-symbolic and symbolic division tasks, a measure of numeral identification and the formal division test.

#### Procedure

Children in both experiments completed all tasks individually with an experimenter in a quiet room at their after-school program. Children completed the non-symbolic and symbolic division tasks first and the order of the two tasks were counterbalanced across children. The order in which all other tasks were administered was random across participants and was dependent on the duration of the task and the child’s availability. The approximate division tasks and the dot comparison task were run in MATLAB and programmed using the Psychophysics Toolbox extension (Brainard, 1997; Pelli, 1997; Kleiner et al., 2007). The approximate division tasks, video of the tasks, and data are available online at OSF (https://osf.io/g5y27/?view_only=0a2ab8862d9d4e95b2426cb116 57e78d).

The non-symbolic and symbolic division tasks and dot comparison task were run on a 15-inch touch screen laptop computer. Children in Experiment 1 completed the division tasks, dot comparison task, Key-Math-3 Numeration subtest, Woodcock-Johnson Reading Cluster, formal division test and numeral identification task for a total of 45-60 min across 2-3 days. Children also completed a math anxiety questionnaire, but these results are not reported in the current manuscript. Children in Experiment 2 completed both division tasks, numeral identification test, and formal division task in one session. All children received stickers to maintain motivation.

### Experimental Tasks

#### Introduction to the Non-Symbolic and Symbolic Division Tasks

Children were introduced to a bee named “Buzz” on the computer screen. The experimenter read the following story: “Buzz flies to flowers to find food to bring back to his hive. Buzz lands on the flower to get the food, and some of the food sticks to him. When Buzz flies away from the flower, some of the food falls down onto the flower.” A picture on the computer screen showed Buzz carrying four dots above a flower with two petals. Four dots fell toward the flower with two dots landing on each petal. The children were told, “The same amount of food falls on each petal of the flower.” The children were then shown Buzz above a flower with four petals and eight dots falling toward the flower. The children were told, “We can see the food falling down toward the flower. See how even if the flower looks different, the same amount of food falls onto each petal.” Children were then shown Buzz above a flower with eight petals and eight dots falling toward the flower. The instructions were repeated one more time. The experimenter never mentioned the number of dots or petals.

#### Non-Symbolic Division

**FIGURE 1:**
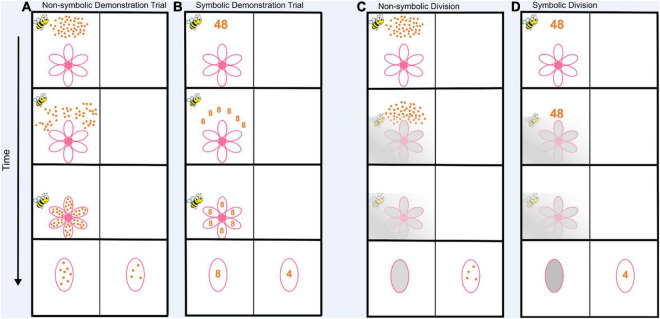
Schematic of the non-symbolic and symbolic division tasks. **(A)** Demonstration trials for the non-symbolic task. Children and adults watched as the dots on the top of the screen fell onto the petals below. Then, one of the flower petals moved toward the center of the left side of the screen and the target petal appeared with a new quantity of dots to compare on the right side of the screen. Participants responded by touching the petal with the greater quantity of dots. Participants completed 8 demonstration trials. The demonstration trials are not included in any analysis. **(B)** The demonstration trials for the symbolic version of the task. Participants watched the numeral at the top of the screen split apart and change into the numerals that landed on the flower at the bottom of the screen. **(C)** Experimental trials for the non-symbolic division task. The task was identical to the demo trials, except that as the dots fell to the bottom of the screen a cloud appeared that obscured how many dots fell onto each petal. Then the obscured petal moved to the middle of the left side of the screen and subjects had to imagine how many dots were on this petal and compare it to the visible target quantity. **(D)** The experimental trials for the symbolic version of the task. Participants watched the dividend numeral move and disappear into the fog without viewing the quotient. Then participants compared their imagined quotient to the new target number on the right side of the screen. For a video of the division tasks please see https://osf.io/g5y27/?view_only=b57c188ca72f4b48a0447fdff1470dc9.

Please see Supplementary videos 1-4 for a video recording of the division tasks. After the initial instructions, the demonstration phase of the game began (Figure 1A). Children watched an animated set of dots fall onto the petals of a flower. On demonstration trials, children could see how many dots fell onto each flower petal (i.e., they saw the result of the division operation). The initial number of dots is the dividend, the number of petals is the divisor, and the number of dots that fall onto one petal is the quotient. After the dots fell onto the flower petals the flower disappeared and one of the petals from the flower moved to the middle of the left side of the screen. A new flower petal with dots already inside appeared on the right side of the screen. The experimenter asked, “Which petal has more food?” The child was told they should touch the petal to indicate their answer. The trial did not progress until the child made their response, but the experimenter encouraged the child to make their choice quickly. Once the child touched a petal, a happy bee with the words “Great job!” appeared for the correct response or a sad bee with the words “try again!” appeared for an incorrect response. Then, a screen appeared with Buzz in the center. The child was told to touch Buzz to continue playing the game. Touching Buzz started the next trial. Children completed eight demonstration trials. The purpose of these trials was to ensure children understood that the same number of dots fall into each petal of the flower, and that their job was to pick the petal that had more food. These trials were not used in any analyses because the result of the division operation was visible to the child on these trials. During the demonstration phase flowers had 2, 5, and 8 petals.

After completing the demonstration phase children were told, “Now it is a foggy day in the garden, but Buzz still needs your help. You won’t have enough time to count all the food Buzz is carrying, and because of the weather, you won’t be able to see the amount of food that falls onto each petal. Instead, you’ll need to *imagine* how many pieces of food are on each petal. Remember, the same amount of food falls onto each petal.” After these instructions, the experimenter started the first experimental trial (Figure 1C). An array of dots and an empty flower appeared on the left side of the screen like before, but this time when the dots began to fall toward the flower a fog appeared over the flower. Children could still see the outline of the flower petals and the dots falling, but the dots were obscured before they were distributed onto the petals. Children could thus no longer see how many dots landed on each flower petal but had to mentally divide the dots by the number of petals to infer the quotient. Like in the demonstration, the flower disappeared and one of the petals moved up to the middle of the left side of the screen. However, this time the inside of the petal was foggy (gray) so that the child could not see the number of dots inside the petal. Another flower petal appeared on the right side of the screen with dots visible inside. Then the experimenter said, ‘‘Ok, which petal has more food? Try and imagine how many pieces of food are on this [gesture to left] petal even though you can’t see them!’’ Children then responded by touching the petal they thought had the greater quantity of dots and received feedback. Children completed 32^1^ trials with automated feedback. During these 32 trials children saw flowers with 2, 5, or 8 petals in random order. To test whether children could generalize to new divisors, after the completion of the first 32 trials children completed 24^2^ more trials with 3 or 6 petals without automated feedback. Throughout all trials the experimenter never mentioned any number words. Note that the animation always occurred on the left side of the screen so that children could anticipate where to attend, however, the correct choice (i.e., larger quantity) was counterbalanced across all trials. Accuracy and reaction time were recorded for each trial.

#### Symbolic Division

The symbolic division task was identical to the non-symbolic version, except that all dividends and comparison targets were displayed with Arabic numerals instead of dots (Figure 1D). The instructions and the numerical values remained identical between both versions of the division tasks. Children received eight demonstration trials at the beginning of the task (Figure 1B). During these trials, the numeral at the top of the screen (dividend) fell toward the flower and split apart into the numeral representing quotients (e.g., the numeral “32” split into four numerals “8” and each numeral “8” fell onto one of the four petals in the above example). Children then completed 32 trials with feedback and 24 trials without feedback. For example, on a given trial a child might see the numeral “32” and the numeral would then float down behind the fog onto a flower with four petals. The child would have to imagine “8” on each petal. If the child were presented with a target comparison petal with the numeral “4” the correct answer would be the foggy petal (8 > 4).

#### Numerical Values for the Division Tasks

We chose numerical values for the approximate division tasks to ensure that children had to pay attention to all three numbers (dividend, divisor, target) to solve the task successfully. All target comparison numbers were drawn from the same values as the possible quotients. The quotients used (8, 10, 13, 17, 22, 29, 37, 48) were chosen to be approximately evenly spaced on a log scale (Supplementary Figure 1). This allowed the ratio between the quotient and the target to be independent of the magnitude of the divisor, dividend and quotient. This is important because the difficulty between comparing any two numerosities is dependent on the ratio between them (Feigenson et al., 2004). By including an equal number of trials at each ratio for each divisor we could compare accuracy at each divisor, and test whether participants could generalize to novel divisors after learning the non-symbolic division task.

Experiment 1 included 32 trials with feedback testing divisors of 2, 5, and 8 and 24 trials without feedback testing divisors of 3 and 6 (Supplementary Table 1). In Experiment 2 children completed 32 trials with feedback testing divisors of 2, 5, and 8 and 24 trials without feedback testing divisors of 3, 4, and 6 (Supplementary Table 2). The numerical values chosen in Experiment 2 ensured that participants would have chance level performance if they chose their answer based on the median target value displayed.

#### Dot Comparison Task

Two dot arrays appeared on a black screen for 750 ms. The arrays were then occluded, and the task was to touch the numerically larger array. Children completed 200 trials with feedback on every trial. The number of dots ranged from 8 to 32. The stimuli were created to evenly sample a stimulus space that varied by the ratio between the number, size, and the spacing of the dots. To encourage greater reliability of the measurement, trial level difficulty was titrated (Lindskog et al., 2013). The titration procedure calculated the percentage correct over the last five trials. The ratio between the two dot arrays moved to an easier ratio if accuracy was 3 out of 5 or less, stayed the same if accuracy was 4 out of 5, and moved to a more difficult ratio if the accuracy was 5 out of 5. A quantitative index of each child’s ANS acuity was calculated as a Weber fraction (*w*) as specified in DeWind et al. (2015). This model accounts for the effects of non-numerical features of dot arrays on numerical discrimination and has been shown to provide more reliable estimates of *w* (DeWind and Brannon, 2016).

#### Numeral Identification Task

The numerals 1-30 were printed and displayed individually on index cards. The numerals were displayed in random order, and the child was asked “What number is this?” Accuracy was recorded. The majority of children in our sample successfully recognized all numerals 1-30 (69 out of 79 participants tested) and so this task was not included in subsequent analyses.

#### Key Math-3 Diagnostic Assessment

The Numeration section of the Key Math-3 Diagnostic Assessment Form B (Connolly, 2007) is a test of general basic math skills like place value, counting, the relative magnitude of numbers. It also tests understanding of fractions, decimals, and percentages. We used the age standardized scale score.

#### Woodcock-Johnson IV Test of Cognitive Abilities

Participants’ reading abilities were assessed using the “Basic Reading Skills” cluster of the Woodcock-Johnson. This cluster is comprised of the “Letter-Word Identification” and “Word Attack” subtests. In the “Letter-Word Identification” subtest, participants named letters and read words aloud. In “Word Attack,” participants read nonsense words and identified letter sounds. We used the age standardized Basic Reading Skills score.

#### Formal Division Test

We created a test of 15 questions that examined children’s addition and division knowledge. Six items were word problems, eight items were symbolic arithmetic problems, and one item required the experimenter to show the child a picture of the division symbol (÷) and ask, “Do you know what this symbol is?” For each arithmetic problem, the child was shown a flashcard with the arithmetic equation as the experimenter read the problem aloud. The test questions are reproduced in Supplementary Table 3. A division knowledge score was calculated based on a child’s accuracy on the four symbolic division problems (range 0-4) and whether or not they could identify the division symbol.

### Experiment 3

#### Subjects

Participants were eighty-seven undergraduates (mean age 20.7 years old, 51 female). Written and informed consent was collected in accordance with a protocol accepted by the University of [blinded] Institutional Review Board. Seven participants did not return to complete the second session and were thus excluded from the mediation analysis. The data from two dot comparison scores and two fraction magnitude comparison scores were lost due to computer error.

#### Procedure

Adults completed all tasks in two sessions that took place on separate days no more than 3 days apart and received course credit as compensation. Testing occurred in a quiet room on a touch screen desktop computer. During the first session adults completed the non-symbolic and symbolic division tasks in counterbalanced order, the vocabulary test, and the division strategy questionnaire. During the second session subjects completed an addition verification task, a dot comparison task, and a fraction magnitude comparison task in counterbalanced order. Subjects completed a math anxiety questionnaire, but this data is not included in the current report.

#### Non-Symbolic and Symbolic Division Tasks

The tasks and instructions for adults were identical to those described for children in Experiment 1. The participants were told that this task was created for use with children to explain the presence of the cartoon bee and storyline.

#### Dot Comparison Task

The task was the same as that described for the children.

#### Vocabulary Test

Subjects answered 42 multiple choice vocabulary questions in 5 min. The questions were taken from the Kit of Factor-Referenced Cognitive Tests (Ekstrom et al., 1976). Performance was calculated as the number of problems answered correctly minus 1/4 of the number incorrect to discourage guessing.

#### Addition Verification Test

One and two digit addition and subtraction problems were displayed horizontally with a proposed answer (e.g., 27 + 52 = 79). Subjects pressed the F or J key (counterbalanced) if the statement was correct and the F or J key if it was incorrect. On incorrect trials (50% of all trials) the sum displayed was ± 10 or ± 2 from the correct sum as modeled after (Klein et al., 2010). Participants had 10 seconds to make a response. Subjects completed two blocks of 96 trials each. Performance was quantified as the median reaction time on correctly rejected incorrect equations.

#### Fraction Magnitude Comparison Task

Subjects viewed two fractions displayed in the middle of the screen in white on a black background. The goal of the task was to pick the fraction greater in magnitude by pressing the F key for the left fraction or the J key for the right fraction. The stimuli were the same as used in Fazio et al. (2015). Accuracy and reaction time were recorded.

#### Division Strategy Questionnaire

The goal of this questionnaire was to examine the strategies adults used to solve the non-symbolic and symbolic division tasks.

The full questionnaire and results are reported in the Supplementary Table 4.

### Analysis Plan

#### Alternative Heuristic Analysis

We conducted a series of analyses to test the possibility that participants were using an alternative heuristic instead of approximately dividing. If participants attempted to compare the divisor (number of petals) to the target comparison number when making their response performance would not exceed chance expectations since the target was greater than the divisor on all trials. Alternatively, participants could attempt to compare only the dividend to the target comparison number. The target was greater than the dividend on only three trials (3/56), and so we confirmed that children and adults performed at above chance levels when excluding those three trials.

We next examined whether participants used a heuristic where they constructed a mental model of the median of the target value across all trials and evaluated whether the target on a given trial was more or less than the median target value. Using this heuristic, subjects would pick the target value if it were greater than the median target value and they would pick the imagined quotient if the target were less than the median target value. The stimulus set used in Experiment 1 was not designed to rule out this alternative strategy, however, the stimulus set constructed for Experiment 2 ensured that participants could not score above chance if they relied on this strategy. Thus, above chance performance in Experiment 2 rules out the possibility that children rely on the median target strategy to solve the approximate division tasks. We tested for use of the median target heuristic in adults by calculating accuracy on the few trials where the median target strategy was ineffective.

#### Mediation Analysis

We ran mediation models to test the hypothesis that non-symbolic division mediates the relationship between ANS acuity and formal mathematics ability. We removed any outlier scores greater or less than three times the interquartile range for children and adults. This process removed four ANS acuity scores from the child dataset, and 3 symbolic division scores from the adult dataset. We used the natural log transformation on both child and adult ANS acuity scores (Child ANS acuity Shapiro-Wilk W = 0.96; adult ANS acuity W = 0.91, W value close to 1 represents a normal distribution). Bivariate correlations and descriptive statistics are reported for children in Experiment 1 in Supplementary Table 5 and adults in Supplementary Table 6. To ensure that correlations between measures were not simply due to age in the children, we partialed out age from our measures of ANS acuity and symbolic and non-symbolic division.

To measure formal math ability we used the Key-Math-3 Numeration subtest in children, and accuracy on the fraction magnitude comparison test in adults. We did not run a mediation model using the Addition Verification measure in adults because this measure was not significantly correlated with ANS acuity (Supplementary Table 6; *r* = −0.14, *p* = 0.21). Mediation analyses test for a significant indirect effect that accounts for some portion of the original direct effect. The goal of this analysis was to examine whether non-symbolic division skill accounts for significant variance in the relation between ANS acuity and symbolic math ability in both children and adults. A significant mediation would be consistent with our hypothesis that non-symbolic division calculation is a mechanism of the relation between ANS acuity and symbolic math, though we cannot test for causality in our cross-sectional design. A significant mediation result in our adult participants would support the idea that non-symbolic calculation skill underlies the small but significant relation between ANS acuity and symbolic math in adults expert in symbolic number (Schneider et al., 2016).

## Results

### Non-Symbolic Division Performance

**FIGURE 2:**
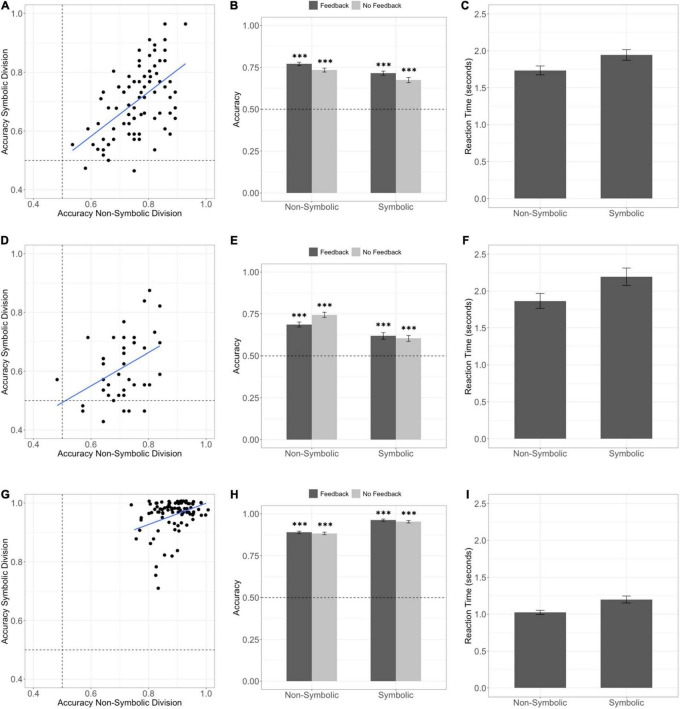
Children and adults can successfully perform approximate division over non-symbolic and symbolic operands. The dotted line depicts chance performance. Error bars depict the standard error of the mean. ^***^p < 0.001 **(A)** Scatter plot depicting approximate non-symbolic and symbolic division overall accuracy in Experiment 1. **(B)** Children in Experiment 1 performed with above chance accuracy on both symbolic and non-symbolic division tasks on trials with feedback. Children also performed significantly above chance on both tasks during the no-feedback phase of the task where participants needed to divide with novel divisors. **(C)** Group level means of median reaction time on correct trials for the non-symbolic and symbolic tasks in Experiment 1. **(D)** Scatter plot depicting approximate non-symbolic and symbolic division overall accuracy in Experiment 2. **(E)** Children in Experiment 2 performed with above chance accuracy on both symbolic and non-symbolic division tasks on trials with feedback. Children also performed significantly above chance on both tasks during the no-feedback phase of the task where participants needed to divide with novel divisors. **(F)** Group level means of median reaction time on correct trials for the non-symbolic and symbolic tasks in Experiment 2. **(G)** Scatter plot depicting approximate non-symbolic and symbolic division overall accuracy in Experiment 3 with adult subjects. Points are jittered horizontally to reveal overlapping data points. **(H)** Adults in Experiment 3 performed with above chance accuracy on both symbolic and non-symbolic division tasks on trials with feedback and trials without feedback with novel divisors. **(I)** Group level means of median reaction time on correct trials for the non-symbolic and symbolic tasks in Experiment 3.

Children and adults performed well above chance expectations on both the feedback (children 77%, t_88_ = 27.4, *p* < 0.001, *d* = 2.9; adults 89%, t_86_ = 60.1, *p* < 0.001, *d* = 6.4) and no feedback (children 73%, t_88_ = 19.8, *p* < 0.001, *d* = 2.1; adults 88%, t_86_ = 51.2, *p* < 0.001, *d* = 5.5) phases of the non-symbolic division task (Figure 2). These data indicate successful generalization of the division operation to novel divisors and demonstrate that both children and adults engaged in approximate division.

In Experiment 2, with stimuli designed to rule out a median target alternative strategy, we replicated the finding that children can perform non-symbolic intuitive division and generalize to novel divisors with a different set of numerical values chosen from the same stimulus space (Figure 2; feedback 69% accuracy t_41_ = 12.8, *p* < 0.001, *d* = 2.0; no feedback 74% accuracy t_41_ = 15.9, *p* < 0.001, *d* = 2.5).

### Symbolic Division Performance

**FIGURE 3:**
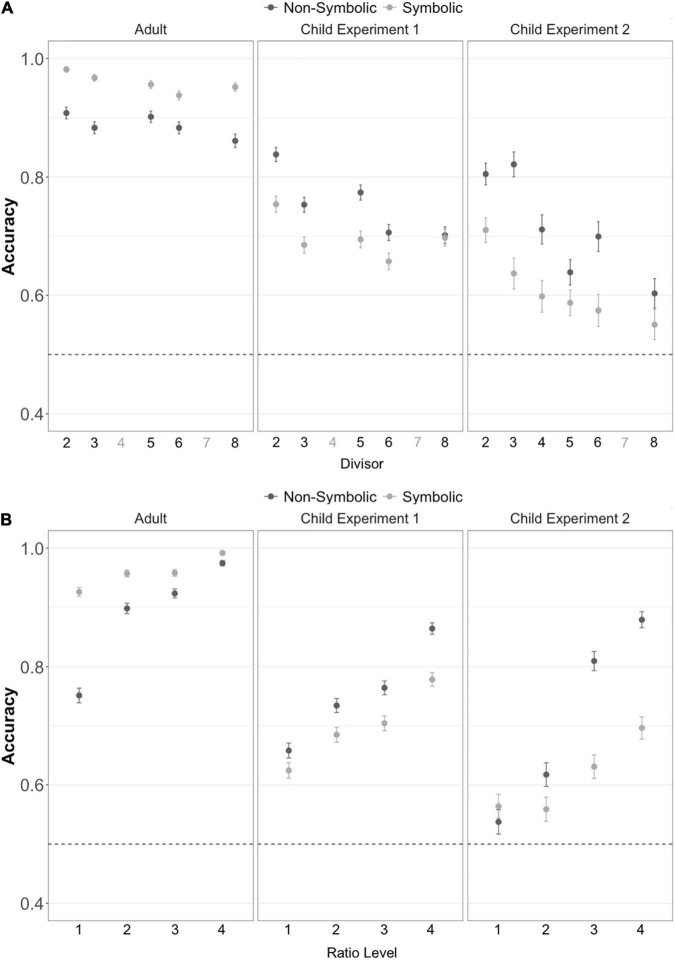
Children and adults can successfully perform approximate division across **(A)** varied divisors and **(B)** ratios between the quotient and comparison quantity. Error bars represent the standard error of the mean. Divisors depicted in gray on the x-axis were not used in the experiment. Ratio level 1 ≈0.8, Ratio level 2 ≈0.6, Ratio level 3 ≈0.45, Ratio level 4 ≈0.35. The dotted line represents chance performance.

Children and adults performed well above chance on both the feedback (children 72%, t_88_ = 17.4, *p* < 0.001, *d* = 1.8; adults 96%, t_86_ = 72.8, *p* < 0.001, *d* = 7.8) and no feedback (children 67%, t_88_ = 11.5, *p* < 0.001, *d* = 1.2; adults 95%, t_86_ = 63.6, *p* < 0.001, *d* = 6.8) phases of the symbolic division task (Figure 3). We replicated this above chance performance with children in Experiment 2 (feedback 62% accuracy t_41_ = 5.99, *p* < 0.001, *d* = 0.92; no feedback 60% accuracy t_41_ = 6.01, *p* < 0.001, *d* = 0.93).

### Adult and Child Division Format Effect

We compared the relative performance of adults and children in Experiment 1 because these experiments were run using the same numerical values. We ran a mixed effects ANOVA predicting overall performance on the division tasks with a main effect of task format (symbolic or non-symbolic) and age group (adult or child), an interaction between format and age, and a random effect of subject. There was a main effect of age group on division performance, (*F*_1_,_174_ = 332.8, *p* < 0.001) and a significant age by task format interaction (*F*_1_,_174_ = 106.1, *p* < 0.001). The main effect indicated that adults performed with higher accuracy overall (t_174_ = 18.2, *p* < 0.001, *d* = 2.8). Follow up tests on the interaction indicated that adults performed with higher accuracy on the symbolic as compared to the non-symbolic version of the division task (paired *t*-test: t_86_ = -10.5, *p* < 0.001, *d* = 1.3), whereas children showed the opposite effect. Children performed significantly better on the non-symbolic version of the task (paired t-test: t_88_ = 5.52, *p* < 0.001, *d* = 0.54). This format effect held even among children who could recognize all the numerals 1-30, indicating that greater accuracy on the non-symbolic task was not due to a lack of basic numeral knowledge (t_78_ = 5.05, *p* < 0.001, *d* = 0.54).

We ran the same analysis on median reaction time on correct trials. There was a main effect of age (F_1_,_174_ = 46.6, *p* < 0.001) driven by the fact that adults were faster than children (1.11 seconds vs. 1.84 seconds t_174_ = 11.0, *p* < 0.001). There was also a significant main effect of task format (*F*_1_,_174_ = 23.6, *p* < 0.001) driven by the fact that both adults and children were faster to perform approximate division on the non-symbolic than the symbolic task (paired *t*-test t_175_ = 4.87, *p* < 0.001; median RT adult non-symbolic = 1.01 s, median RT adult symbolic = 1.10 s, median RT non-symbolic child = 1.62 s, median RT symbolic child = 1.81 s). There was no significant format by age interaction (*F*_1_,_174_ = 0.194, *p* = 0.66).

### Effect of Divisor on Division Accuracy

In Experiment 1, children performed significantly above chance (50%) on the non-symbolic division task independently for all divisors tested (divisor 2 t_88_ = 25.8, *p* < 0.001, *d* = 2.7; divisor 3 t_88_ = 19.3, *p* < 0.001, *d* = 2.0; divisor 5 t_88_ = 22.9, *p* < 0.001, *d* = 2.4; divisor 6 t_88_ = 14.9, *p* < 0.001, *d* = 1.6; divisor 8 t_88_ = 13.5, *p* < 0.001, *d* = 1.4). In Experiment 2, children also performed significantly above chance (50%) on the non-symbolic division task independently for all divisors tested (divisor 2 t_41_ = 13.0, *p* < 0.001, *d* = 2.0; divisor 3 t_41_ = 11.9, *p* < 0.001, *d* = 1.8; divisor 4 t_41_ = 9.29, *p* < 0.001, *d* = 1.4; divisor 5 t_41_ = 7.32, *p* < 0.001, *d* = 1.3; divisor 6 t_41_ = 10.2, *p* < 0.001, *d* = 1.6; divisor 8 t_41_ = 4.81, *p* < 0.001, *d* = 0.74). The same pattern of results was found in Experiment 3 with adult subjects (divisor 2 t_86_ = 41.5, *p* < 0.001, *d* = 4.4; divisor 3 t_86_ = 38.8, *p* < 0.001, *d* = 4.2; divisor 5 t_86_ = 41.6, *p* < 0.001, *d* = 4.5; divisor 6 t_86_ = 37.8, *p* < 0.001, *d* = 4.0; divisor 8 t_86_ = 29.1, *p* < 0.001, *d* = 3.1).

On the symbolic division tasks, children and adults also performed with above chance accuracy on all divisors independently, with the exception of the divisor 8 in Experiment 2 where participants performed marginally above chance (Experiment 1 divisor 2 t_88_ = 14.1, *p* < 0.001, *d* = 1.5; divisor 3 t_88_ = 11.2, *p* < 0.001, *d* = 1.2; divisor 5 t_88_ = 13.5, *p* < 0.001, *d* = 1.4; divisor 6 t_88_ = 8.79, *p* < 0.001, *d* = 0.93; divisor 8 t_88_ = 11.6, *p* < 0.001, *d* = 1.2; Experiment 2 divisor 2 t_41_ = 7.87, *p* < 0.001, *d* = 1.2; divisor 3 t_41_ = 5.16, *p* < 0.001, *d* = 0.80; divisor 4 t_41_ = 3.71, *p* < 0.001, *d* = 0.57; divisor 5 t_41_ = 3.07, *p* = 0.004, *d* = 0.47; divisor 6 t_41_ = 2.44, *p* = 0.02, *d* = 0.38; divisor 8 t_41_ = 1.85, *p* = 0.07, *d* = 0.29; Experiment 3 divisor 2 t_86_ = 90.8, *p* < 0.001, *d* = 9.7; divisor 3 t_86_ = 74.2, *p* < 0.001, *d* = 8.0; divisor 5 t_86_ = 54.4, *p* < 0.001, *d* = 5.8; divisor 6 t_86_ = 46.8, *p* < 0.001, *d* = 5.0; divisor 8 t_86_ = 50.1, *p* < 0.001, *d* = 5.4). These results indicate that above chance accuracy on the division tasks was not dependent on any single divisor. Thus participants were able to divide across multiple divisors flexibly.

#### Effect of the Ratio Between the Target and Quotient on Division Accuracy

**FIGURE 4:**
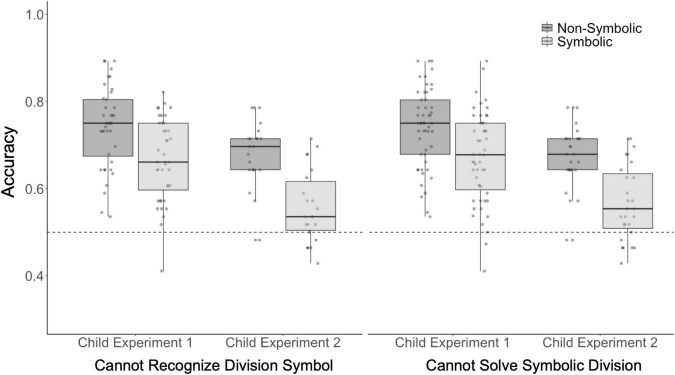
Children who cannot recognize the division symbol (÷) or cannot solve symbolic division are able to perform non-symbolic and symbolic approximate division. Children’s performance on the non-symbolic and symbolic division tasks broken down by their performance on the formal division test. The feedback and no feedback trials are combined for each task. Boxes indicate median and first and third quartiles. The whiskers indicate 1.5 multiplied by the interquartile range from the first and third quartiles. The dotted line represents chance performance. Each point reflects one participant’s accuracy.

To test whether accuracy was dependent on the ratio between the quotient and the comparison target value, we fit a generalized linear mixed effects model (GLMM) following a binomial error distribution predicting whether each trial was correct with the ratio between the quotient and target as a fixed effect and a random effect of subject. Six models were fit, one for each of the three experiments and two task formats. For the non-symbolic division task this model indicated significant main effects of ratio for both adults and children in both Experiments 1 and 2 (Figure 4 adult β = 0.18, *z* = 3.28, *p* = 0.001; child experiment 1 β = 0.62, *z* = 13.8, *p* < 0.001; child experiment 2 β = 0.34, *z* = 5.95, *p* < 0.001). For the symbolic division task this model also indicated significant main effects of ratio for both adults and children in both experiments (Figure 4; adult β = 0.33, *z* = 3.37, *p* < 0.001; child experiment 1 β = 0.55, *z* = 13.5, *p* < 0.001; child experiment 2 β = 0.41, *z* = 7.67, *p* < 0.001).

#### Effect of Formal Division Knowledge on Approximate Division

**FIGURE 5:**
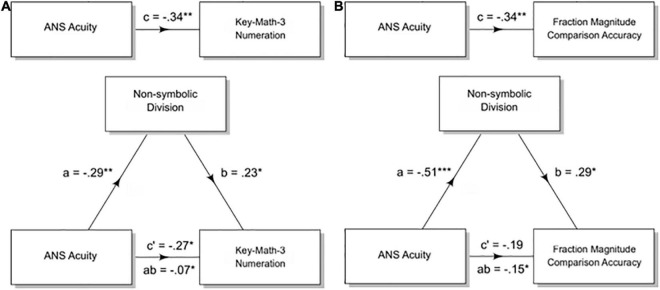
Non-symbolic division mediates the effect of ANS acuity on symbolic math abilities in adults and children. Mediation analyses test for a significant indirect effect (the product of the standardized coefficients a and b) that accounts for some portion of the original direct effect (c). The remaining direct effect is represented as c’. The models in this figure test whether non-symbolic division performance mediates the relation between ANS acuity and a measure of formal math skills in children (Key-Math-3 Numeration) and adults (Fraction Magnitude Comparison). **(A)** Non-symbolic division accuracy partially mediates the relation between ANS acuity and a child’s score on the Key-Math-3 Numeration section. Both the indirect (ab) and the direct path c’ are significant. **(B)** Non-symbolic division accuracy fully mediates the relation between ANS acuity and accuracy on the fraction magnitude comparison test. The remaining direct effect (c’) is no longer significant, while the indirect effect (ab) is significant as tested with a bootstrap estimate approach. * < 0.05, ** < 0.01, *** < 0.001.

There were 40 children who could not identify the division symbol in Experiment 1. Children who could not identify the division symbol successfully completed both the non-symbolic and symbolic division tasks with above chance accuracy (Figure 5; non-symbolic 74% t_39_ = 16.5, *p* < 0.001, *d* = 2.6; symbolic 67% t_39_ = 11.2, *p* < 0.001, *d* = 1.8). In Experiment 2, we replicated the finding that children who could not identify the division symbol nevertheless performed at above chance level on our approximate division tasks (non-symbolic 68% t_21_ = 11.6, *p* < 0.001, *d* = 2.5; symbolic 56% t_21_ = 3.31 *p* = 0.003, *d* = 0.71).

There were 51 children who could not solve any of the four simple symbolic division problems on our formal division test in Experiment 1 (for example, 6 ÷ 3 = ?). These simple division problems were read aloud so that children who could not recognize the ÷ symbol could rely on the spoken word ‘divided’ to solve these problems. Children who could not solve symbolic division problems were nevertheless significantly above chance on both approximate division tasks (Figure 5; non-symbolic 74% t_50_ = 19.0, *p* < 0.001, *d* = 2.7; symbolic 67% t_50_ = 11.7 *p* < 0.001, *d* = 1.6). In Experiment 2 we replicated these results (non-symbolic 68% t_26_ = 13.1, *p* < 0.001, *d* = 2.5; symbolic 57% t_26_ = 4.06 *p* < 0.001, *d* = 0.78). Together, these data indicate that formal knowledge of division is not necessary to solve the approximate division tasks, in either symbolic or non-symbolic format.

For a comparison of approximate division accuracy between children who did and did not demonstrate formal division knowledge please see the Supplementary Material.

#### Alternative Heuristic Analysis

Adults and children in Experiments 1 and 2 performed with above chance accuracy on both the non-symbolic and symbolic approximate division tasks when excluding the trials where subjects could compare the dividend and the target to get the correct answer, indicating participants did not rely on this heuristic to complete the division tasks (adults non-symbolic t_86_ = 62.4, *p* < 0.001; adults symbolic t_86_ = 71.1, *p* < 0.001; children experiment 1 non-symbolic 75% t_88_ = 27.3, *p* < 0.001; children experiment 1 symbolic 69% t_88_ = 15.1, *p* < 0.001; children experiment 2 non-symbolic t_41_ = 16.2, *p* < 0.001; children experiment 2 symbolic 60% t_41_ = 6.32, *p* < 0.001).

We next examined whether performance was consistent with participants creating a mental model of the median target value to solve the division task. The stimulus set in Experiment 2 with children was designed to rule out this heuristic, and as reported above, children performed with above chance accuracy on both the symbolic and non-symbolic division tasks in Experiment 2 (Figures 2, 3). This indicates children can solve our approximate division task without use of a median target heuristic. The stimulus set used by adult subjects was not designed to rule out this heuristic, however, the accuracy rate was above chance on the subset of trials that could not be solved using the median target heuristic (non-symbolic 64%, t_86_ = 6.64, *p* < 0.001; symbolic 91%, t_86_ = 28.4, *p* < 0.001).

#### Mediation Analysis

For children, ANS acuity was a significant predictor of a participant’s score on the Key-Math-3 Numeration subtest (standardized β = −0.34, *p* = 0.002) and of accuracy on the non-symbolic division task (standardized β = −0.29, *p* = 0.009). ANS acuity continued to be a significant predictor of the score on the Numeration subtest after controlling for the mediator, non-symbolic division accuracy, however the strength of this relation was lessened (ANS acuity standardized β = −0.27, *p* = 0.02; non-symbolic division accuracy standardized β = 0.23, *p* = 0.04). We tested the significance of this reduction using a non-parametric bootstrap estimation with 5,000 simulations of the indirect effect using the “mediation” package in R (Tingley et al., 2014; indirect effect = −0.07, 95% CI = [−0.17 −0.01], *p* = 0.03). The direct effect was also significant, indicating a mediation (direct effect = −0.27, 95% CI = [−0.48.05], *p* = 0.02). The proportion mediated was 0.20 (*p* = 0.03, 95% CI = [0.01.69]). Thus, sharper ANS acuity was associated with 0.07 standard deviations higher Key-Math-3 Numeration score as mediated through non-symbolic division accuracy (Figure 5A). This finding is in line with our hypothesis, however, when we partialed out the relation between the Woodcock-Johnson Reading Cluster and the Numeration subtest, ANS acuity was no longer significantly correlated with scores on the Numeration subtest (ANS acuity standardized β = −0.18, *p* = 0.13). This indicates that the relation between ANS acuity and the Numeration subtest is not specific to math skills, but rather to general academic performance.

For adults, ANS acuity was a significant predictor of accuracy on the fraction magnitude comparison test (standardized β = −0.34, *p* = 0.003) and of accuracy on the non-symbolic division task (standardized β = −0.51, *p* < 0.001). ANS acuity was no longer a significant predictor of accuracy on the fraction magnitude test after controlling for the mediator, non-symbolic division accuracy (ANS acuity standardized β = −0.19, *p* = 0.12; non-symbolic division accuracy standardized β = 0.29, *p* = 0.02). Non-symbolic division accuracy mediated the relation between ANS acuity and accuracy on the fraction magnitude comparison test. The indirect effect was significant when tested with a bootstrap estimation approach with 5,000 simulations (indirect effect = −0.15, 95% CI = [−0.28, −0.03], *p* = 0.01). The direct effect was not significant, indicating a mediation (direct effect = −0.19, 95% CI = [-0.4 6, 0.07], *p* = 0.16). The proportion mediated was 0.43 (95% CI = [0.08, 1.5]), *p* = 0.02. Thus, sharper ANS acuity was associated with 0.15 standard deviations higher fraction magnitude comparison accuracy as mediated through non-symbolic division accuracy (Figure 5B). The indirect effect remained significant when controlling for the relation between a participants’ vocabulary score and fraction magnitude comparison accuracy (indirect effect = −0.15, 95% CI = [−0.29, −0.03], *p* = 0.009, direct effect = −0.19, 95% CI = [−0.46, 0.08], *p* = 0.17, proportion mediated = 0.44, CI = [08, 1.6], *p* = 0.02).

## Discussion

The current experiments are the first to demonstrate that elementary school children and adults can approximately divide over both non-symbolic arrays and numerals. Our task required a true non-symbolic, approximate division computation that integrates the relations between a dividend, divisor, and quotient. Successful completion of the two division tasks was not dependent on formal knowledge of division. Children who could not recognize the division symbol nor solve simple division problems were nevertheless successful at performing non-symbolic division, and more surprisingly, they were also able to complete the division task when the dividend and target comparison number were represented symbolically with Arabic numerals. These findings highlight the depth of intuitive math knowledge that children possess before formal education.

We found that task format differentially impacted children and adult’s division accuracy. Whereas adults were significantly better at the symbolic compared to the non-symbolic division task, children were significantly better at the non-symbolic task. The timing of when symbols facilitate more accurate arithmetic calculations may mark an important conceptual milestone in mathematical development. One possibility is that making a switch to more accurate computation within the symbolic number system earlier in development is a better scaffold for increasingly complex computation. Alternatively, continuing to root a mathematical operation in its underlying concrete representation may be a better foundation for understanding complex math concepts. Future research can test whether the timing of this transition is longitudinally predictive of later math achievement, how other characteristics of the learner impact a child’s non-symbolic and symbolic arithmetic accuracy over time, and whether instructional practices can impact the timing of this transition.

One exciting implication of these findings is the possibility to introduce the division concept early in math education via large number approximate calculation using both non-symbolic quantities and numerals. Future research can explore whether an explicit linking between non-symbolic division and division using numerals can aid formal division understanding. The theoretical framework of concreteness fading may be a particularly useful method for implementing such an intervention (Fyfe et al., 2015; Fyfe and Nathan, 2018). A progression from practice with approximate non-symbolic division, to approximate symbolic division to exact symbolic division may be a way to link children’s intuitions about division to formal division knowledge. Another theoretical framework that has shown promise in linking intuitive math knowledge to symbolic math learning is implicit analogical transfer (Sidney and Thompson, 2019). Under this framework, ‘warming up’ activities are used to promote successful analogical transfer between current and future knowledge. In the context of the current findings, intuitive division tasks could be used to activate children’s intuitive understanding of a division topic before a lesson in formal division. Under both frameworks, grounding abstract arithmetic concepts in children’s intuitive understanding of arithmetic may boost children’s conceptual understanding of arithmetic operations and their confidence in their own skill to perform such calculations. Incorporating numerical symbols within an intuitive division context may function as a pedagogical bridge for developing a deeper and more robust division concept in children, which ultimately could promote stronger symbolic, exact division calculation skill.

The current experiments also examined whether approximate division could be a mechanism of the known relation between ANS acuity and symbolic mathematics (Schneider et al., 2016). Two pieces of evidence strongly suggest that the ability to non-symbolically and approximately divide is grounded in the Approximate Number System. First, accuracy for all subjects was modulated by the ratio between the target and quotient in both non-symbolic and symbolic format, indicating use of an approximate strategy when making their choice. Ratio dependent discrimination of quantity is a hallmark of the ANS (Feigenson et al., 2004). Second, accuracy on both division tasks was significantly correlated with participant’s ANS acuity as independently measured with a dot comparison task. The division operation joins a growing number of mathematical operations that can be represented using the ANS before formal math education including addition, subtraction, scaling, ratio comparison, and solving for X (Barth et al., 2005; McCrink and Wynn, 2007; Kibbe and Feigenson, 2015; McCrink et al., 2016).

With this evidence that approximate division is rooted in the ANS, we then tested the second aspect of our hypothesis – that approximate division ability is correlated with symbolic math skill. In line with our hypothesis, non-symbolic division mediated the relation between ANS acuity and symbolic math in both children and adults. Sharper ANS acuity may facilitate greater accuracy in a student’s conceptual model of a division operation, and this conceptual model may function as a scaffold for formal symbolic computation. Thus, the mechanism for the established link between ANS acuity and symbolic math ability may be rooted in the computational abilities allowed by the ANS, and not in the acuity of the ANS *per se*. Having a strong mental model of what it means to divide (or engage in other operations such as subtraction or multiplication) may in turn create a strong foundation for the learning of abstract mathematical concepts. The significant mediation effect in adults suggests that adults continue to use approximate mental models to calculate, even once they have knowledge of exact calculation techniques.

Unexpectedly, in children, while non-symbolic division ability was a mediator of the relation between ANS acuity and Key-Math-3 performance, this mediation effect was no longer significant when controlling for the correlation between children’s scores on the Woodcock-Johnson Reading Cluster and their performance on the Key-Math-3 Numeration test. When controlling for reading ability, ANS acuity was no longer correlated with scores on the Key-Math-3. It is possible this result is due to shared correlations between an unknown additional skill, such as inhibition or executive function (Fuhs and McNeil, 2013), and our measures of math, reading, and ANS acuity. However, we do not interpret this finding as evidence that ANS acuity is meaningfully related to reading skill, but rather as evidence of the strong correlation between math and reading skills is typical in children of this age that is attributed to extrinsic academic factors (Wang et al., 2015; Cantin et al., 2016). In the current study, we found a correlation of r = 0.62 between Key-Math-3 and Woodcock Johnson Reading Cluster scores after controlling for age of the participants. This strong correlation between math and reading scores left little variance to partition in the mediation model. In adults, partialing out vocabulary skill from fraction magnitude accuracy (*r* = 0.02) did not impact our mediation effect. Thus, this unexpected finding is most likely due to measurement rather than theoretical importance of reading skill in the relation between the ANS and symbolic math. Indeed, we do not find this pattern of results in the adult experiment.

In conclusion, our study highlights that children have strong intuitive math abilities before they begin formal math education. We found that children are remarkably good at dividing large numbers regardless of whether they were presented non-symbolically or symbolically, and this ability is not limited to simply halving or quartering. Children’s extraordinary success at approximate division with large quantities suggests the possibility that introducing non-symbolic arithmetic calculation early in math education may be beneficial for formal arithmetic learning. We hope that our findings inspire future studies to test the efficacy of math instruction that emphasizes grounding highly abstract mathematical concepts in intuitive math abilities.

## Data Availability Statement

The datasets presented in this study can be found in online repositories. The names of the repository/repositories and accession number(s) can be found below: https://osf.io/g5y27/?view_only=0a2ab8862d9d4e95b2426cb11657e78d.

## Ethics Statement

The studies involving human participants were reviewed and approved by University of Pennsylvania Human Subjects Electronic Research Application (HS-ERA). Written informed consent to participate in this study was provided by the participants’ legal guardian/next of kin.

## Author Contributions

ES, HZ, ND, and EB designed the study. ES and HZ wrote the code to run the experimental tasks and collected the data. ES analyzed the data and wrote the first draft of the manuscript. All authors edited the final manuscript.

## Conflict of Interest

The authors declare that the research was conducted in the absence of any commercial or financial relationships that could be construed as a potential conflict of interest.

## Publisher’s Note

All claims expressed in this article are solely those of the authors and do not necessarily represent those of their affiliated organizations, or those of the publisher, the editors and the reviewers. Any product that may be evaluated in this article, or claim that may be made by its manufacturer, is not guaranteed or endorsed by the publisher.

## Footnotes

1 11 subjects ran a longer version of the division tasks (53 trials with feedback and 40 trials without feedback) but the number of trials for the remaining subjects was reduced due to time constraints at the after-school programs where testing occurred.

2 One subject ran 9 extra trials without feedback due to a computer malfunction. Two subjects ran a majority (19 or 20 out of 24) but not all 24 trials due to an early pick up time. These subjects are included in the analysis.
