# Supplementary material for: Young Children Intuitively Divide Before They Recognize the Division Symbol
Source: Front Hum Neurosci. 2022 Feb 25;16:752190. doi: 10.3389/fnhum.2022.752190 (PMC8913505; doi:10.3389/fnhum.2022.752190)
Supplement: Supplementary file 1 [file Data_Sheet_1.docx]

Supplementary Material

**All supplementary videos can be accessed in the Approximate Division Task Videos folder at:**

<https://osf.io/g5y27/?view_only=b57c188ca72f4b48a0447fdff1470dc9>

**Supplementary Video 1.** Nonsymbolic Demo.mp4. This is a video recording of demonstration trials of the non-symbolic division task.

**Supplementary Video 2.** Nonsymbolic Trials.mp4. This is a video recording of feedback and no feedback trials of the non-symbolic division task.

**Supplementary Video 3.** Symbolic Demo.mp4. This is a video recording of demonstration trials of the symbolic division task.

**Supplementary Video 4.** Symbolic Trials.mp4. This is a video recording of feedback and no feedback trials of the symbolic division task.


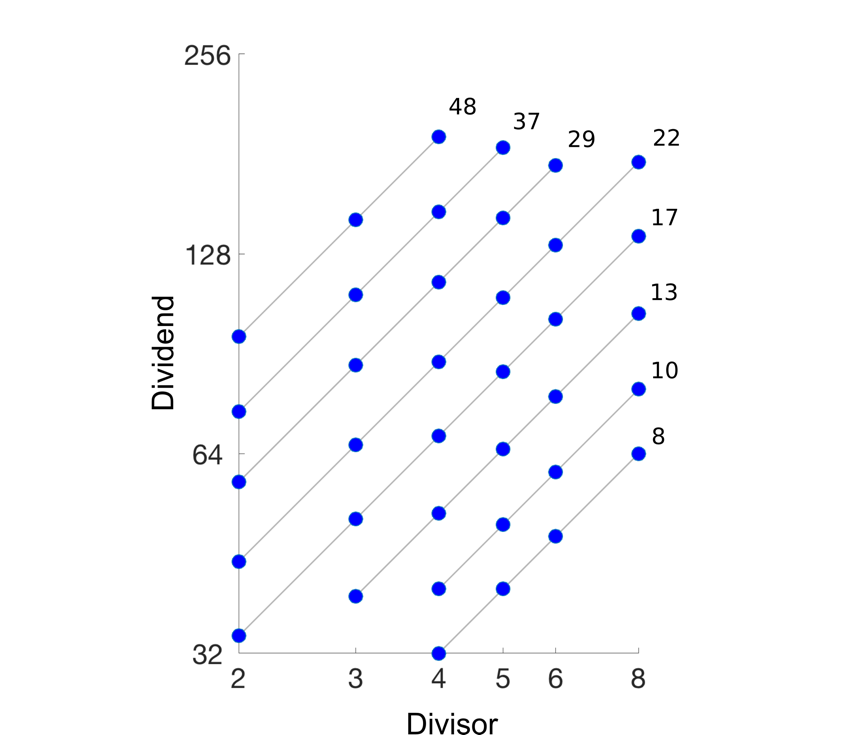


**Supplementary Figure 1. The divisor, dividend and quotients used in all experiments represented as a stimulus space.** The divisor is the number of petals on the flower and the dividend is the number of dots that appeared at the top of the screen at the beginning of a trial. Each diagonal line represents a given quotient (the quantity in one petal). The quotients were chosen to be approximately evenly separated in log space such that the ratio between two neighboring quotients corresponds to roughly the same ratio. The same values (8, 10, 13, 17, 22, 29, 37 and 48) were used for the target comparison values that appeared on the right side of the screen. The quotient was the correct choice on 50% of trials.

Supplementary Table 1. Experiment 1 stimuli for both children and adults.

| Dividend | Divisor | Target | Ratio Level | Phase |
| --- | --- | --- | --- | --- |
| 14 | 2 | 25 | N/A | Demo |
| 45 | 5 | 3 | N/A | Demo |
| 24 | 2 | 3 | N/A | Demo |
| 75 | 5 | 4 | N/A | Demo |
| 40 | 8 | 18 | N/A | Demo |
| 24 | 8 | 11 | N/A | Demo |
| 25 | 5 | 18 | N/A | Demo |
| 48 | 8 | 2 | N/A | Demo |
| 44 | 2 | 29 | 1 | feedback |
| 85 | 5 | 22 | 1 | feedback |
| 145 | 5 | 37 | 1 | feedback |
| 80 | 8 | 13 | 1 | feedback |
| 44 | 2 | 17 | 1 | feedback |
| 110 | 5 | 17 | 1 | feedback |
| 65 | 5 | 10 | 1 | feedback |
| 136 | 8 | 13 | 1 | feedback |
| 44 | 2 | 37 | 2 | feedback |
| 65 | 5 | 22 | 2 | feedback |
| 104 | 8 | 22 | 2 | feedback |
| 136 | 8 | 29 | 2 | feedback |
| 44 | 2 | 13 | 2 | feedback |
| 110 | 5 | 13 | 2 | feedback |
| 136 | 8 | 10 | 2 | feedback |
| 176 | 8 | 13 | 2 | feedback |
| 34 | 2 | 37 | 3 | feedback |
| 44 | 2 | 48 | 3 | feedback |
| 40 | 5 | 17 | 3 | feedback |
| 136 | 8 | 37 | 3 | feedback |
| 74 | 2 | 17 | 3 | feedback |
| 58 | 2 | 13 | 3 | feedback |
| 110 | 5 | 10 | 3 | feedback |
| 136 | 8 | 8 | 3 | feedback |
| 34 | 2 | 48 | 4 | feedback |
| 65 | 5 | 37 | 4 | feedback |
| 64 | 8 | 22 | 4 | feedback |
| 136 | 8 | 48 | 4 | feedback |
| 74 | 2 | 13 | 4 | feedback |
| 185 | 5 | 13 | 4 | feedback |
| 145 | 5 | 10 | 4 | feedback |
| 176 | 8 | 8 | 4 | feedback |
| 66 | 3 | 29 | 1 | no feedback |
| 102 | 6 | 22 | 1 | no feedback |
| 60 | 6 | 13 | 1 | no feedback |
| 51 | 3 | 13 | 1 | no feedback |
| 144 | 3 | 37 | 1 | no feedback |
| 102 | 6 | 13 | 1 | no feedback |
| 39 | 3 | 22 | 2 | no feedback |
| 51 | 3 | 29 | 2 | no feedback |
| 102 | 6 | 29 | 2 | no feedback |
| 111 | 3 | 22 | 2 | no feedback |
| 78 | 6 | 8 | 2 | no feedback |
| 174 | 6 | 17 | 2 | no feedback |
| 66 | 3 | 48 | 3 | no feedback |
| 132 | 6 | 48 | 3 | no feedback |
| 78 | 6 | 29 | 3 | no feedback |
| 66 | 3 | 10 | 3 | no feedback |
| 51 | 3 | 8 | 3 | no feedback |
| 174 | 6 | 13 | 3 | no feedback |
| 39 | 3 | 37 | 4 | no feedback |
| 51 | 3 | 48 | 4 | no feedback |
| 60 | 6 | 29 | 4 | no feedback |
| 87 | 3 | 10 | 4 | no feedback |
| 174 | 6 | 10 | 4 | no feedback |

Supplementary Table 2. Stimuli for Experiment 2 with children.

| Dividend | Divisor | Target | Ratio Level | Phase |
| --- | --- | --- | --- | --- |
| 14 | 2 | 25 | N/A | Demo |
| 45 | 5 | 3 | N/A | Demo |
| 24 | 2 | 3 | N/A | Demo |
| 75 | 5 | 4 | N/A | Demo |
| 40 | 8 | 18 | N/A | Demo |
| 24 | 8 | 11 | N/A | Demo |
| 25 | 5 | 18 | N/A | Demo |
| 48 | 8 | 2 | N/A | Demo |
| 58 | 2 | 48 | 2 | feedback |
| 58 | 2 | 17 | 2 | feedback |
| 74 | 2 | 22 | 2 | feedback |
| 34 | 2 | 37 | 3 | feedback |
| 44 | 2 | 48 | 3 | feedback |
| 74 | 2 | 17 | 3 | feedback |
| 96 | 2 | 22 | 3 | feedback |
| 34 | 2 | 48 | 4 | feedback |
| 96 | 2 | 17 | 4 | feedback |
| 58 | 2 | 10 | 4 | feedback |
| 74 | 2 | 13 | 4 | feedback |
| 40 | 5 | 10 | 1 | feedback |
| 50 | 5 | 13 | 1 | feedback |
| 145 | 5 | 22 | 1 | feedback |
| 85 | 5 | 13 | 1 | feedback |
| 40 | 5 | 13 | 2 | feedback |
| 85 | 5 | 29 | 2 | feedback |
| 145 | 5 | 17 | 2 | feedback |
| 185 | 5 | 22 | 2 | feedback |
| 110 | 5 | 48 | 3 | feedback |
| 185 | 5 | 17 | 3 | feedback |
| 145 | 5 | 13 | 3 | feedback |
| 85 | 5 | 48 | 4 | feedback |
| 64 | 8 | 10 | 1 | feedback |
| 80 | 8 | 13 | 1 | feedback |
| 176 | 8 | 17 | 1 | feedback |
| 80 | 8 | 8 | 1 | feedback |
| 64 | 8 | 13 | 2 | feedback |
| 136 | 8 | 37 | 3 | feedback |
| 104 | 8 | 37 | 4 | feedback |
| 136 | 8 | 48 | 4 | feedback |
| 176 | 8 | 8 | 4 | feedback |
| 40 | 4 | 13 | 1 | no feedback |
| 60 | 6 | 13 | 1 | no feedback |
| 32 | 4 | 13 | 2 | no feedback |
| 48 | 6 | 13 | 2 | no feedback |
| 87 | 3 | 37 | 1 | no feedback |
| 39 | 3 | 10 | 1 | no feedback |
| 40 | 4 | 8 | 1 | no feedback |
| 87 | 3 | 48 | 2 | no feedback |
| 78 | 6 | 8 | 2 | no feedback |
| 66 | 3 | 48 | 3 | no feedback |
| 102 | 6 | 37 | 3 | no feedback |
| 132 | 6 | 48 | 3 | no feedback |
| 39 | 3 | 37 | 4 | no feedback |
| 51 | 3 | 48 | 4 | no feedback |
| 102 | 6 | 48 | 4 | no feedback |
| 132 | 6 | 8 | 4 | no feedback |
| 174 | 6 | 22 | 1 | no feedback |
| 111 | 3 | 22 | 2 | no feedback |
| 148 | 4 | 22 | 2 | no feedback |
| 144 | 3 | 22 | 3 | no feedback |
| 148 | 4 | 17 | 3 | no feedback |
| 192 | 4 | 22 | 3 | no feedback |
| 192 | 4 | 17 | 4 | no feedback |
| 192 | 4 | 17 | 4 | no feedback |

Supplementary Table 3. *Formal Division Test*

**Script: Division Questions**

1. Do you know what 2 plus 3 is?
2. If Sam has four apples and Kate gives two more how many apples will he have?
3. Do you know what half of 4 is?
4. How about half of 18?
5. What if you ordered a pizza and it had 8 slices? If there were 4 of you who wanted to share the pizza how many slices would you each get?
6. The Football Factory makes 49 footballs per week. If the factory is open 7 days a week, how many footballs do they make per day?
7. Do you know what this symbol is? ÷

Can you solve any of these problems?

6 + 3 = ?

8 + 2 = ?

5 + 5 = ?

12 + 4 = ?

6÷3 = ?

8 ÷ 4 = ?

24÷3 = ?

45÷5 = ?

Supplementary Table 4. *Adult Division Strategy Questionnaire*

1. How did you solve the task with the bee, flowers, and DOTS? Did you use any specific strategies?
2. How did you solve the task with the bee, flowers, and NUMBERS? Did you use any specific strategies?
3. Which task was more difficult, the flowers & bees task with DOTS or with NUMBERS?
4. How many petals appeared on a flower? (circle all that you remember)

1 2 3 4 5 6 7 8 9 10 11 12

1. What mathematical operation did the task with the flowers and bees test?
2. What differences were there between parts 1 and parts 2 of both flowers and bees games?
3. Which strategy best describes how you solved the task with the bee, flowers, and DOTS? (Circle the answer that best describes your strategy)
4. I got a sense of the amount of dots on the top of the screen and the amount of petals below, and imagined approximately how many dots were on one petal.
5. I got a sense of the amount of dots on the top of the screen and counted the number of petals and imagined approximately how many dots were on one petal.
6. I estimated the number of dots on the top of the screen and assigned a number to my estimate. I counted the number of flower petals. Then I calculated using those numbers.
7. Other, please explain ______________________________________________________________________
8. Which strategy best describes how you solved the task with the bee, flowers, and NUMBERS? (Circle the answer that best describes your strategy)
9. I looked at the number up top and estimated the number of petals below, and imagined approximately how many dots were on one petal.
10. I looked at the number up top and counted the number of petals and imagined approximately how many dots were on one petal.
11. I calculated the exact answer using the number up top and the exact number of petals.
12. Other, please explain ______________________________________________________________________

**Division Strategy Questionnaire Analysis.**

These analyses focus on questions 3, 4, 7 and 8. Thirty subjects completed a shorter version of the questionnaire without questions 3 and 4, and so they are not included in the analysis of those questions. Consistent with the accuracy data reported above, the majority of adult subjects indicated that the non-symbolic version of the division task was more difficult than the symbolic version (43/57). On the non-symbolic task, the majority of participants (47/85, 2 choose not to answer) reported using an approximate strategy by choosing the option “*I got a sense of the amount of dots on the top of the screen and the amount of petals below, and imagined approximately how many dots were on one petal*” on question 7. In contrast, on the symbolic task the majority of participants (50/85) reported using an exact calculation strategy by picking the choice “*I calculated the exact answer using the number up top and the exact number of petals*” on question 8. Of the 34 participants who reported exact calculation on the symbolic task and also completed question 4, only about half could correctly identify the divisors (2,3,5,6,8) used during the tasks (18/34).

Supplementary Table 5. *Descriptive statistics and bivariate correlation matrix of the child data in Experiment 1*

|  |  | *M* | *SD* | 1 | 2 | 3 | 4 |
| --- | --- | --- | --- | --- | --- | --- | --- |
| 1 | Non-symbolic division | .755 | .08 |  |  |  |  |
| 2 | Symbolic division | .710 | .12 | .52*** |  |  |  |
| 3 | ANS acuity | .336 | .15 | -.30** | -.20 |  |  |
| 4 | Key-Math-3 Numeration | 10.0 | 3.6 | .37*** | .32** | -.34** |  |
| 5 | Reading Cluster | 99.4 | 15 | .32** | .22* | -.35** | .62*** |

*Note*. *M* = mean, *SD* = standard deviation. The bivariate correlations are controlling for age. These values are uncorrected for multiple comparisons. ANS acuity is calculated from the dot comparison test. The non-symbolic and symbolic division measures is the total accuracy across the feedback and no feedback trials. Note: *** p <.001 ** p < .01 * p < .05

Supplementary Table 6. *Descriptive statistics and bivariate correlation matrix of the adult data*

|  |  | *M* | *SD* | 1 | 2 | 3 | 4 | 5 |
| --- | --- | --- | --- | --- | --- | --- | --- | --- |
| 1 | Non-symbolic division | .887 | .05 |  |  |  |  |  |
| 2 | Symbolic division | .958 | .04 | .34** |  |  |  |  |
| 3 | ANS acuity | .162 | .04 | -.50*** | -.29* |  |  |  |
| 4 | Fraction Comparison Acc | 166 | 17 | .37*** | .45*** | -.34** |  |  |
| 5 | Addition Correct Rejections RT (sec) | 2.03 | .68 | .01 | -.18 | -.14 | .18 |  |
| 6 | Vocabulary | 14.8 | 7.3 | .19 | .01 | -.33** | .02 | .01 |

*Note*. *M* = mean, *SD* = standard deviation. ANS acuity is calculated from the dot comparison test. The non-symbolic and symbolic division measures is the total accuracy across the feedback and no feedback trials. The symbolic division score was highly skewed due to the near ceiling performance on this task (Shapiro Wilk W = .70), and so this measure is not used in any correlational analysis. These values are uncorrected for multiple comparisons. Note: *** p <.001 ** p < .01 * p < .05

**Comparison of Approximate Division Accuracy by Formal Division Knowledge**

There were 40 children who could not identify the division symbol, and 38 children who could identify the division symbol in Experiment 1. There was no significant difference in non-symbolic division accuracy between these two groups (t_76_ = 1.83, p = .07, d = .41), but children who could recognize the division symbol had significantly higher symbolic division accuracy than children who could not (t_76_ = 3.23, p = .002, d = .73). In Experiment 2, children who could recognize the division symbol performed with higher accuracy than children who could not on both tasks (non-symbolic t_37_ = 2.21, p = .03, d = .71; symbolic t_37_ = 4.16, p < .001, d = 1.3).

There were 51 children who could not solve simple division facts, and 30 children who could solve at least one simple division fact in Experiment 1. Children who could solve at least one division fact had significantly higher accuracy on both the non-symbolic and symbolic division tasks (non-symbolic t_79_ = 2.30, p = .02, d = .53; symbolic t_79_ = 2.60, p = .01, d = .60). In Experiment 2, children who could solve at least one division fact performed with higher accuracy than children who could not on both tasks (non-symbolic t_37_ = 4.00, p < .001, d = 1.4; symbolic t_37_ = 4.97, p < .001, d = 1.7).
